# Technical note: The UF/MSK pediatric mesh-based computational human phantom library: applications to organ dosimetry in computed tomography

**DOI:** 10.1088/1361-6560/ae4a81

**Published:** 2026-03-12

**Authors:** Jared M Baggett, Robert J Dawson, Laura E Dinwiddie, Wyatt W Smither, Yitian Wang, Stefan K Wehmeier, Trung Tran, Shreya Pathak, Sean J Domal, Cameron Kofler, Chansoo Choi, Yeon Soo Yeom, Lukas M Carter, Juan C Ocampo Ramos, Pat B Zanzonico, Adam L Kesner, Wesley E Bolch

**Affiliations:** 1 Medical Physics Program College of Medicine, University of Florida, Gainesville, FL, United States of America; 2 J. Crayton Pruitt Family Department of Biomedical Engineering, University of Florida, Gainesville, FL, United States of America; 3 Department of Radiology and Imaging Sciences, Indiana University, Indianapolis, IN, United States of America; 4 Department of Radiation Oncology, University of Texas Southwestern Medical Center, Dallas, TX, United States of America; 5 Department of Radiology, University of Chicago, Chicago, IL, United States of America; 6 Department of Radiation Convergence Engineering, Yonsei University, Wonju, Republic of Korea; 7 Department of Medical Physics, Memorial Sloan Kettering Cancer Center, New York, NY, United States of America; 8 Department of Imaging Physics, University of Texas MD Anderson Cancer Center, Houston, TX, United States of America

**Keywords:** computed tomography, organ dosimetry, pediatric computational phantoms

## Abstract

**Objective.:**

To develop a mesh-based computational human phantom library of pediatric males and females that encompass the body size distributions as seen in the current U.S. population.

**Approach.:**

Using the mesh-type pediatric reference computational phantoms (MRCPs) of International Commission on Radiological Protection (ICRP) Publication 156 as a starting point, the pediatric University of Florida/Memorial Sloan-Kettering (UF/MSK) phantoms were constructed with standing heights (SHs), total body masses (TBMs), and secondary anthropometric parameters representative of the United States population. Body morphometry targets were derived from data published by the Centers for Disease Control and Prevention. The phantoms were created using a combination of in-house Python scripting and manual adjustment of the reference models’ subcutaneous fat layer. A corresponding version of the library with phantoms in an ‘arms up’ position appropriate for computed tomography dosimetry simulation was also constructed.

**Main results.:**

A large library of 153 male and 131 female pediatric mesh-type computational human phantoms was created in this study. For the males, SH ranged from 95 to 185 cm and the TBM ranged from 10 to 125 kg. For the females, SH ranged from 95 to 175 cm and the TBM ranged from 10 to 115 kg. These 284 phantoms, developed from the ICRP 5 year, 10 year, and 15 year male and female MRCPs, are representative of US children within the age range of 2–18 years. An accompanying NIT (newborn-infant-toddler) series—59 males and 59 females—provides a unique phantom library for patients under the age of 2 years down to birth. Representative organ doses are shown across samples of the pediatric UF/MSK phantom library for the Canon Aquilion One Genesis scanner under fixed tube current imaging.

## 1. Introduction

Organ dosimetry is essential to the application of ionizing radiation in medicine, informing clinical practice in diagnostic imaging and enabling continued innovation in cancer radiotherapy. Proper dosimetry is of heightened importance for patients at elevated stochastic risk, such as children and adolescents (Kutanzi et al 2016). Computational human phantoms have long been used in radiation studies to estimate absorbed organ doses in pediatric patient populations, but the sparse availability of detailed representative models has hindered such data collection. While phantom libraries have been developed using stylized or voxel-based technologies to represent various human populations, few pediatric computational human phantom libraries in fully mesh-type formats are available. Examples of polygon mesh-type phantom libraries of children include those presented by Geyer et al (2014), Segars et al (2015), Xie et al (2017), Dahal et al (2025). At present, only one pediatric tetrahedral mesh-type library has been reported (Kim et al (2024).

Recently, the International Commission on Radiological Protection (ICRP) has released in its Publication 156 the next generation of state-of-the-art models termed mesh-type reference computational phantoms (MRCPs) for pediatric subjects (ICRP 2024). These phantoms are comprised of polygon mesh surfaces, allowing modeling of fine tissue layers and non-uniform scaling of body regions. Mesh phantoms can also be tetrahedralized for Monte Carlo (MC) simulations, improving computational performance over voxel phantoms (Yeom et al 2014), avoiding stair-step artifacts, and enabling modeling of thin stem cell targets for dosimetry. These advantages motivated the creation of a new pediatric library representing U.S. children across a wide range of body morphometries for uses in dosimetric studies.

As described in ICRP Publication 156 (ICRP 2024), the ICRP pediatric MRCPs include models for the male and the female newborn, 1 year-old, 5 year-old, 10 year-old, and 15 year-old. Although these ten anatomic models adequately portray the fine details of human anatomy, they cannot sufficiently represent the entirety of pediatric patients regarding standing height (SH), total body mass (TBM), and body shape. The goal of this study was thus to generate a new phantom library using these MRCPs. Given the differences in the available data and construction process, to be discussed later, the MRCPs have been divided into two unique sub-libraries. The newborn and 1 year-old MRCPs were used to create the NIT (Newborn, Infant, Toddler) sub-library, while the 5-, 10-, and 15 year MRCPs were used for non-NIT phantoms (2–18 years). Combined, they form the University of Florida/Memorial Sloan-Kettering (UF/MSK) mesh-based computational pediatric library. A complementary adult UF/MSK library was developed using the ICRP adult MRCPs described in Publication 145 (ICRP 2020, Dawson et al 2025). The construction methodology used to assemble the pediatric UF/MSK phantom library adopts many of the techniques used to construct the adult library and thus the reader is referred to the Dawson *et al* publication for further details.

## 2. Methods

### 2.1. Targeted anthropomorphic parameters for phantoms beyond early childhood ages

The process for determining anthropomorphic targets in non-NIT phantoms followed that of the adult UF/MSK library (Dawson et al 2025). The Centers for Disease Control and Prevention (CDC) collects body morphometry data for pediatric patients in the age range of 2–18 years and publishes their data within their National Health and Nutrition Examination Survey (NHANES). As in the adult library, the phantoms in the UF/MSK pediatric library each have associated primary parameters to include SH and TBM, as well as secondary parameters to include sitting height and circumferences of the upper arm, thigh, calf and buttocks. SH values were determined based on the 5th–95th percentile range for pediatrics and were then parsed into 5 cm bins. Similarly, TBM targets were calculated by parsing the 5th–95th percentile range into 5 kg bins. Additional phantoms outside the NHANES 5th–95th percentile range were added to ensure complete coverage of phantoms in the predecessor UF/NCI library. Secondary parameters were calculated using linear regressions based on the ratio of primary parameters. More information on this approach is found in section 2.1 of Dawson et al (2025).

Unlike in the adult library, multiple MRCPs per sex were used to construct the current pediatric library. As previously mentioned, the non-NIT library was derived from the 5 year, 10 0year and 15 year MRCPs. Each of the MRCPs were either three-dimensionally scaled upward (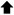) or downward (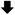) based on the target SH in comparison to the reference phantom SH. The decision to use a particular MRCP to generate phantoms of a given SH was predicated on the difference between the target SH and the SH of the MRCP. Table 1 shows which MRCP model was used for each targeted SH series in the non-NIT pediatric UF/MSK phantom library.

### 2.2. Modeling for non-NIT phantoms

The modeling and construction of the non-NIT pediatric library followed the methodology established for the UF/MSK adult phantom library (Dawson et al 2025). The construction process used an in-house Python script to three-dimensionally scale MCRPs to targeted sitting heights, followed by a one-dimensional scaling of the lower body region to reach the final SH. Residual soft tissue (RST) vertices were adjusted in the graphics software Rhinoceros 3D (www.Rhino3d.com) and Blender 3D (www.blender.org) to match target TBM and secondary parameters. The three outer skin layers (exterior surface and interfaces at 50 *μ*m and 100 *μ*m depth) were generated by outward offsets of the RST layer and any mesh-to-mesh intersections resulting from these offsets were manually corrected to produce the finalized phantom geometry.

As with the adult models, certain cases required manipulation of the base MRCP model prior to scaling and morphing to avoid unwanted intersections. For higher body mass phantoms, outward expansion of the torso and thighs could produce intersections with the arms or between the thighs. To resolve this issue, alternative versions of the base MRCP models were constructed with the limbs rotated outward in 5° increments while preserving internal structure volumes, enabling construction of larger-mass models without mesh collisions. Conversely, for lower body mass phantoms, the inward adjustment of the RST layer resulted in intersections with internal anatomy. In such instances, the underlying organs and structures in the torso were two-dimensionally reduced in 5% increments to accommodate the target mass. Further details of graphical editing of the MRCP models are discussed in section 2.2 of Dawson et al (2025). For the phantoms in the pediatric library derived from the 15 year female MRCP, both the glandular and adipose breast tissues were uniformly scaled to specific volumes as a function of the body mass index (BMI) of the phantom as described in section 2.3 of Dawson et al (2025).

### 2.3. Anthropomorphic parameters for NIT phantoms

The NIT library used the newborn and 1 year MRCPs to model infants 0–24 months. The CDC has published growth charts for infants, containing information related to SH, body mass, and head circumferences^9^. These measurements are categorized by monthly intervals and further expand each month into several percentile groups. However, the CDC data does not include measurements of sitting height for these infants. Consequently, and to maintain a height-scaling process consistent with the non-NIT phantoms, sitting height values for each month were instead extracted from a University of Western Ontario infant autopsy report (Evetts 2016, Evetts et al 2016). Based on these publications, primary parameters were SH and TBM, and secondary parameters were sitting height and head circumference. It is important to note that all values for the selected parameters were sex-averaged, and thus both male and female models were constructed for each collection of targeted parameters.

The phantoms of the NIT series were modeled to represent patient ages in increments of two months at the 10th, 50th and 90th percentile for both SH and TBM at each age point. Additional combinations of phantom SH and TBM were added to the library to produce a finer transition of body morphometries through each percentile group. The exact values for each parameter of these additional models, referred to as the ‘extension’ phantoms, were linearly interpolated or extrapolated from one of the three percentile groups.

### 2.4. Modeling for NIT phantoms

For this library, the newborn MRCP was used as the reference model for all 0 month, 2 month, and 4 month-old phantoms, as well as for the first three extension phantoms. The 1 year-old MRCP was then used for the construction of phantoms representative of 6–24 month models as well as all remaining extension models. The first step of the construction process for phantoms derived from the 1 year MRCP was similar to that of the non-NIT phantoms as an in-house Python script was used to nonuniformly scale the MRCP to match target standing and sitting heights. Using Blender 3D, all interior structures and skin layers (surface, 50 *μ*m, and 100 *μ*m) in the head region were then 2-dimensionally scaled in order to match the outer skin surface to the desired head circumference. Manual adjustment of mesh vertices and faces was then performed on the three skin layers and RST layer, primarily in the torso region, to increase or decrease adipose tissue volume in order to match targeted body mass values.

**Figure 1.**
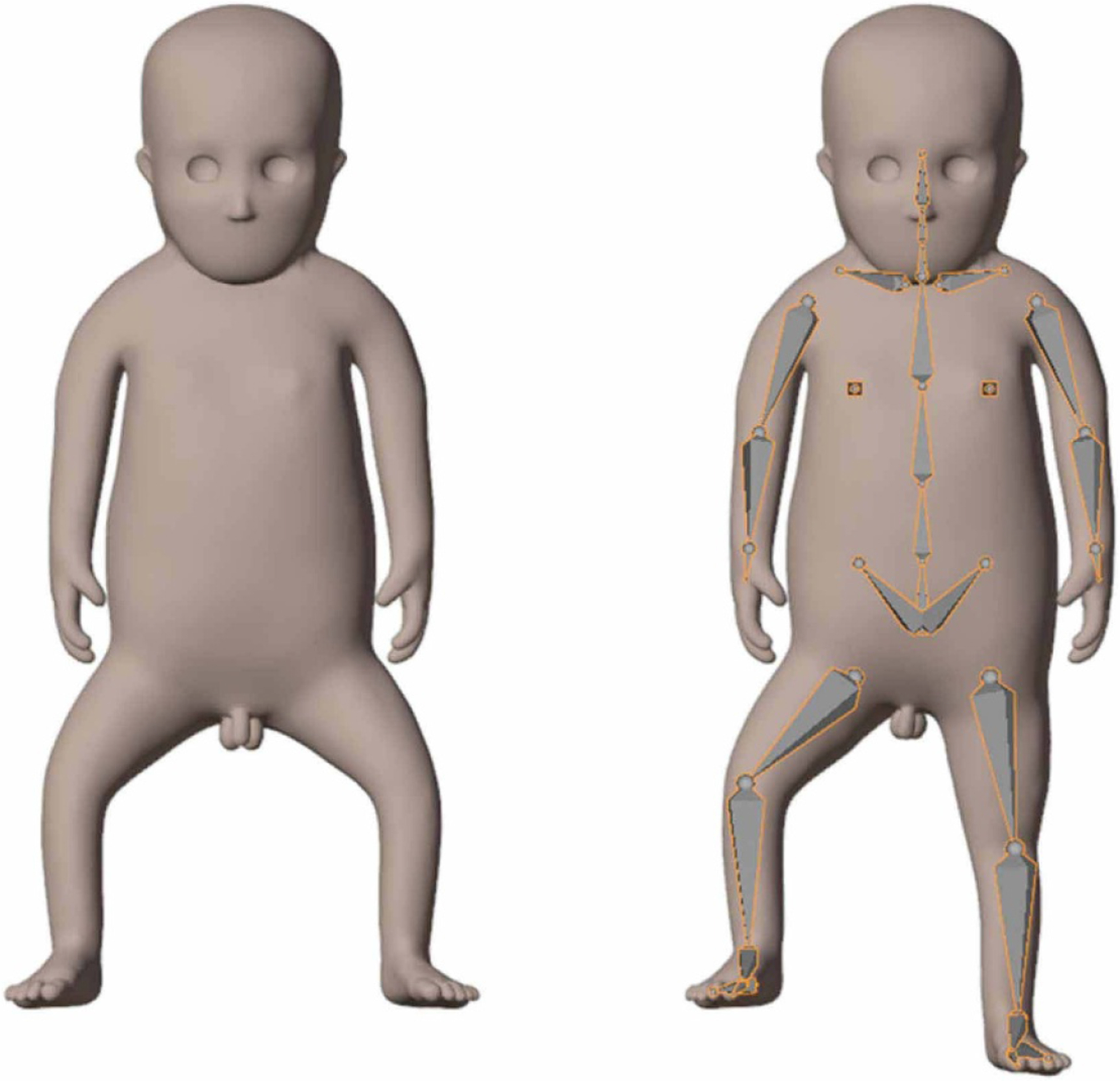
Process of straightening the legs in the newborn male MRCP in Blender 3D. The initial position of the MRCP (left) was adjusted by mapping an anthropomorphic rig (outlined in orange) over boney structures within the phantom and then moving the rig to a straight leg position while conserving the initial volume of the outer skin layer (right).

To more accurately represent the anatomy of a very young infant, the newborn MRCP was originally designed to have its legs bent at both the hip and knee. This body posture can be traced back to the original newborn CT cadaver scan as outline in Nipper et al (2002). Since all other MRCPs have straight legs, an additional step was required to match SH for phantoms constructed using this newborn reference model. First, an anthropomorphic rig was created and aligned with the skeleton of the newborn MRCP in a similar fashion to the rigs created for phantom limb rotation as described in Dawson et al (2025). The legs of the newborn were then straightened to determine the MRCP’s theoretical SH, as would have been measured as part of the NHANES survey. A visualization of this process can be seen in figure 1. After each phantom was three-dimensionally scaled to match exact sitting height, the ratio of the newborn MRCP sitting height to SH was then applied to one-dimensionally scale the legs to achieve the target SH.

### 2.5. Arms up positioning

A significant application of the pediatric library is the construction of a slice-specific organ dose library for computed tomography (CT). For certain clinical CT scans, patients are required to move their arms above their head in an ‘arms up’ (AU) position as to remove them from the field of view. For newborns and very young children, this arms up position is ensured using an extremity constraining device, and thus AU versions of all NIT and non-NIT phantoms were created. This placement of the arms can potentially alter organ dosimetry as compared to the default positioning with the arms by the phantoms’ side, referred to as the ‘arms down’ (AD) position. This issue implied the need for a congruent pediatric library with phantoms in the AU position. To accomplish this, the rigging process used to straighten the infant legs mentioned in the previous section as well as described in section 2.4 of Dawson et al (2025) was utilized. Vertices in the base MRCPs were weighted according to underlying anatomical structures (bone, soft tissue, vessels, etc) so that arm rotations produced anatomically realistic deformations. Each MRCP was adjusted to have its arms repositioned above the head while preserving the volumes of all organ and tissue structures to within 0.1% of the original values. These repositioned phantoms were then height scaled as described previously to generate ‘anchor’ phantoms for each height series. Following this height scaling, the construction process was analogous to that of the adults whereby the RST region for each phantom in the AD position was aligned with the internal organ and tissue structures in the newly repositioned and height-scaled AU anchor phantom. The RST layer (which at this point was of the appropriate height and morphometry metrics, but in the AD position) was then paired with the associated anthropomorphic rig and deformed into the AU position. Multiple mesh offset operations were then performed on the newly modified RST layer to generate the three outer skin layers. Any intersections resulting from these deformations were then resolved manually in Blender.

### 2.6. CT simulations and dosimetry

With the construction of the pediatric phantoms, MC radiation transport simulations were performed on tetrahedral mesh versions of each phantom using a user defined CT source term in the Particle and Heavy Ion Transport code System (PHITS) in order to begin generating a comprehensive slice-specific CT organ dose library. The source term was derived from measurements acquired on a Canon Aquilion ONE Genesis scanner, including half-value layers, lateral dose profiles, beam penumbra, free-in-air kerma at isocenter, and CTDI values measured across combinations of tube potentials, bowtie filters, and collimation widths. Validation of the source model was achieved by reproducing the measured CTDI values in PHITS, with agreement within 5% of the physical measurements. Further details of the CT source term are given in Dawson et al (2025). Using pediatric protocols for CT scans provided by the University of Florida Department of Radiology Standard Names for Imaging Procedures (UF SNIPs)^10^, total organ dose values were calculated by the summation of slice-specific values across a scan range. The exact equations, methods, and source term employed are described in detail in sections 2.5 and 2.6 in Dawson et al (2025). An initial set of organ doses was computed using phantoms derived from the 15 year male MRCP model at 170 cm height for both the AD and AU positions. The simulations were performed using a technique factor combination of a 120 kVp tube potential, a 20 mm collimation, and a medium-sized bowtie filter.

## 3. Results

### 3.1. Height and body mass distributions for final pediatric library

**Figure 2.**
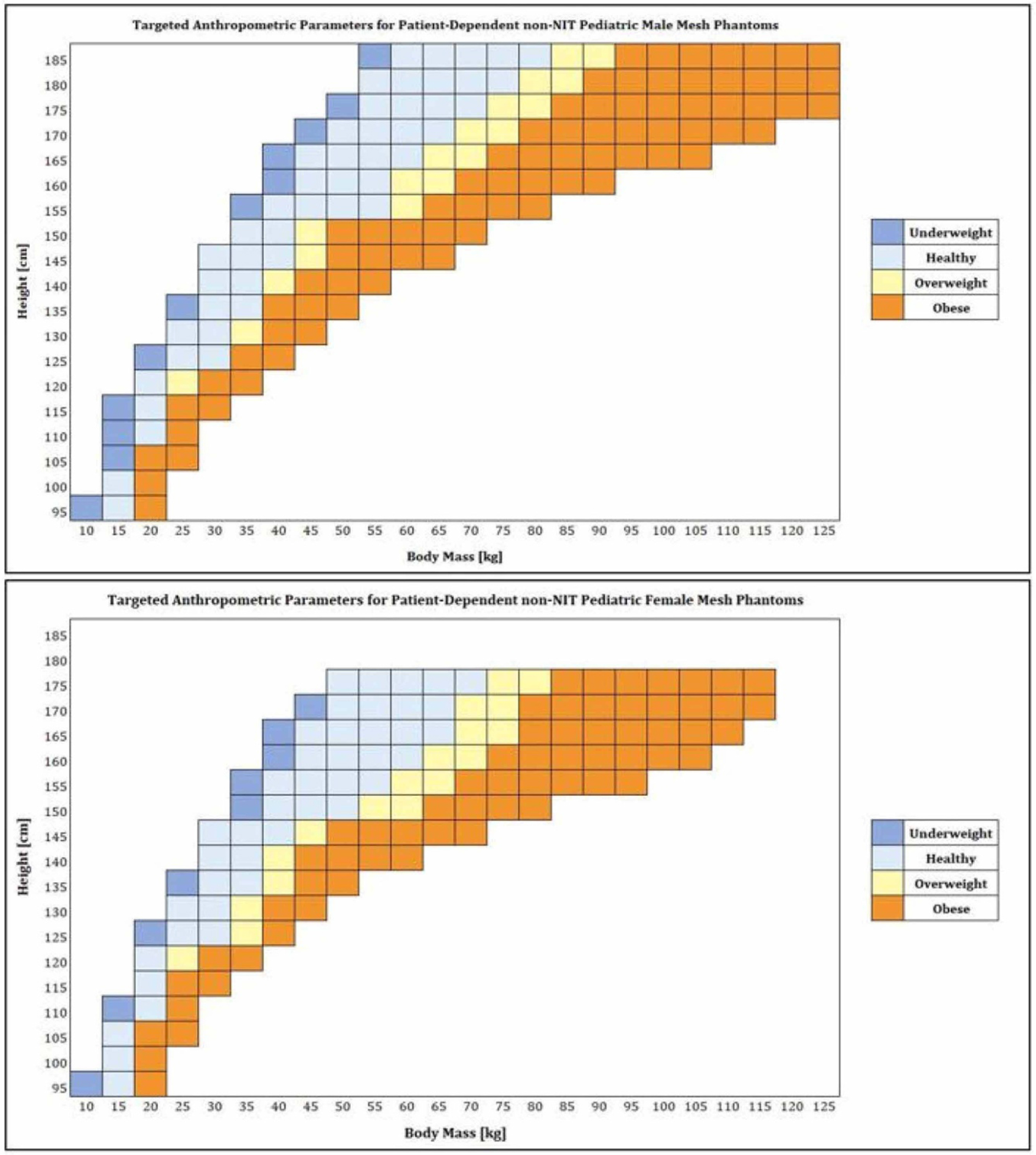
Final standing height and total body mass distributions for non-NIT pediatric male (top) and female (bottom). Body size classifications are based on BMI categories as defined by the CDC.

**Figure 3.**
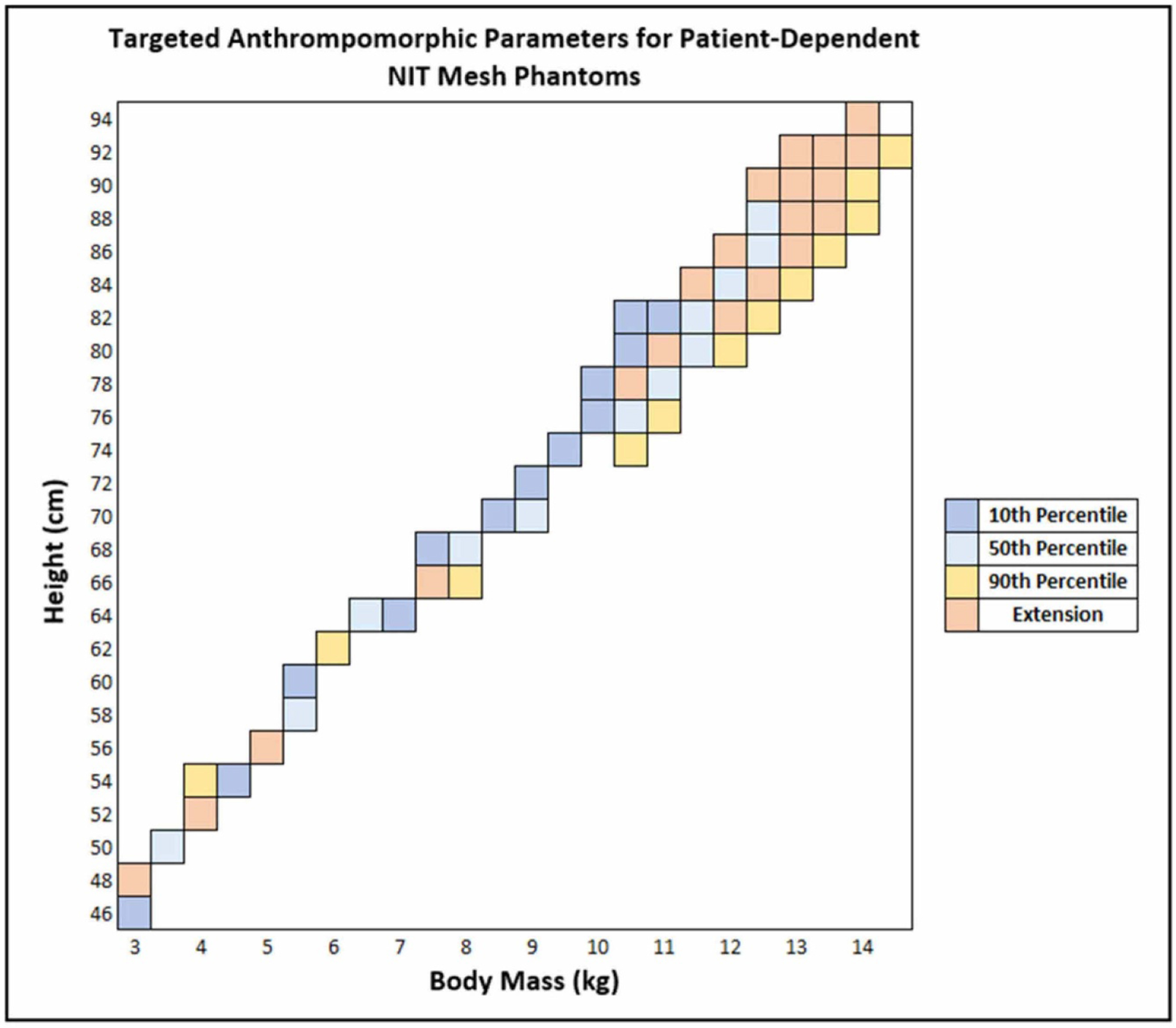
Final standing height and total body mass distributions for all NIT phantoms. Non-extension phantoms are categorized by national average percentiles for standing height and total body mass at a given age. Due to the binning of the axes, not all standing height and total body mass combinations are shown. As such, there are a few instances where different phantom ages and percentile values yield nearly identical values of standing height and total body mass.

In total, 153 male and 131 female non-NIT phantoms were created as well as 59 male and 59 female NIT phantoms. Figures 2 and 3 show the distributions of height and body mass for the non-NIT and NIT phantoms, respectively. All standing and sitting heights were matched to their targets and phantom body masses to within ±1% of their desired values. Annexes A–C provide all tabulated values of the primary and secondary body morphometry targets of pediatric males, pediatric females, and NIT male/female phantoms of the UF/MSK library, respectively. Annexes D–F provide corresponding tabulations of organ masses for these same series of phantoms within the pediatric UF/MSK library.

### 3.2. Polygon mesh models

**Figure 4.**
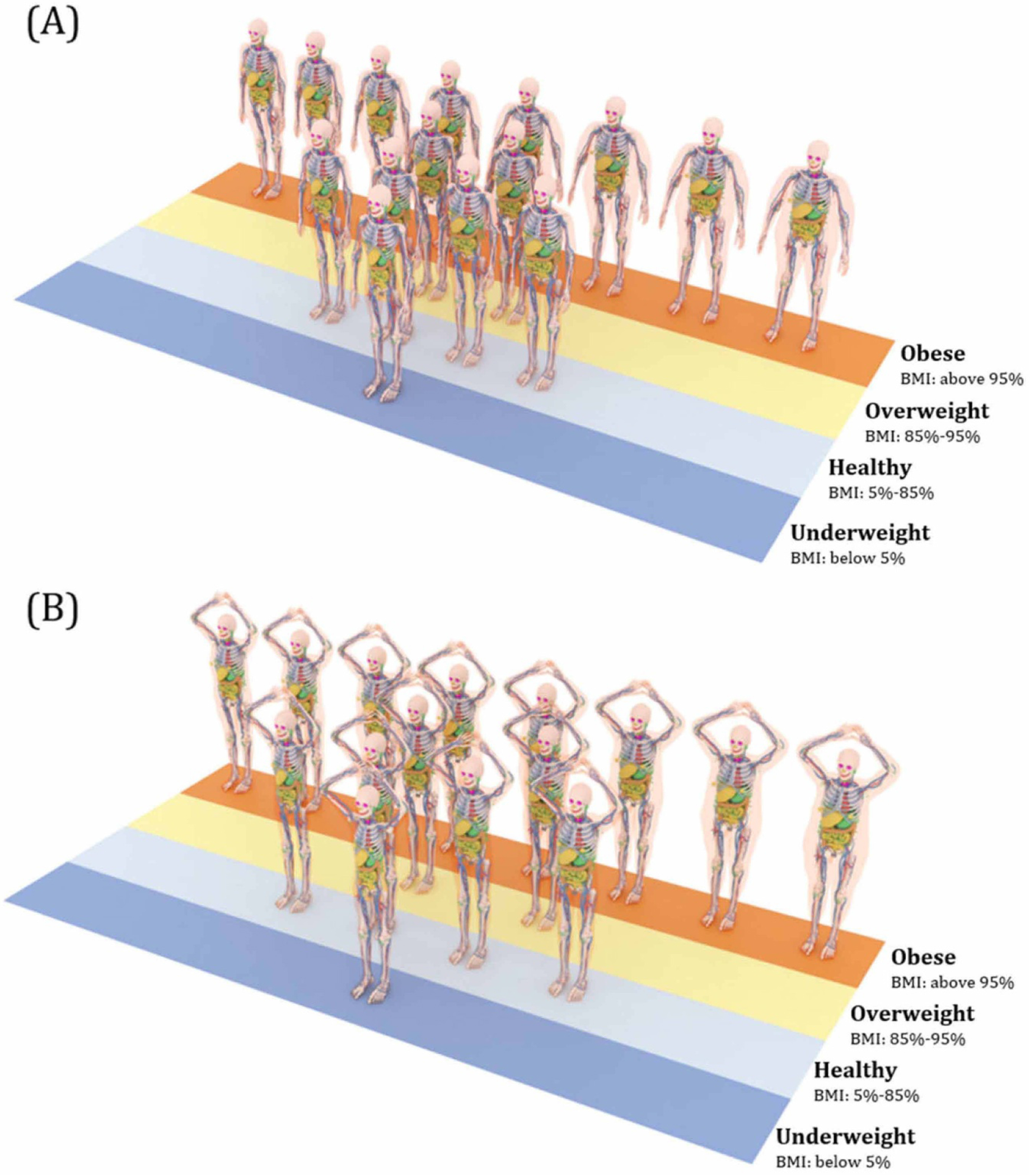
Visualizations of pediatric male phantoms at 170 cm standing height at varying height in the (A) AD (B) AU positions. Phantoms are sorted in rows by BMI category.

**Figure 5.**
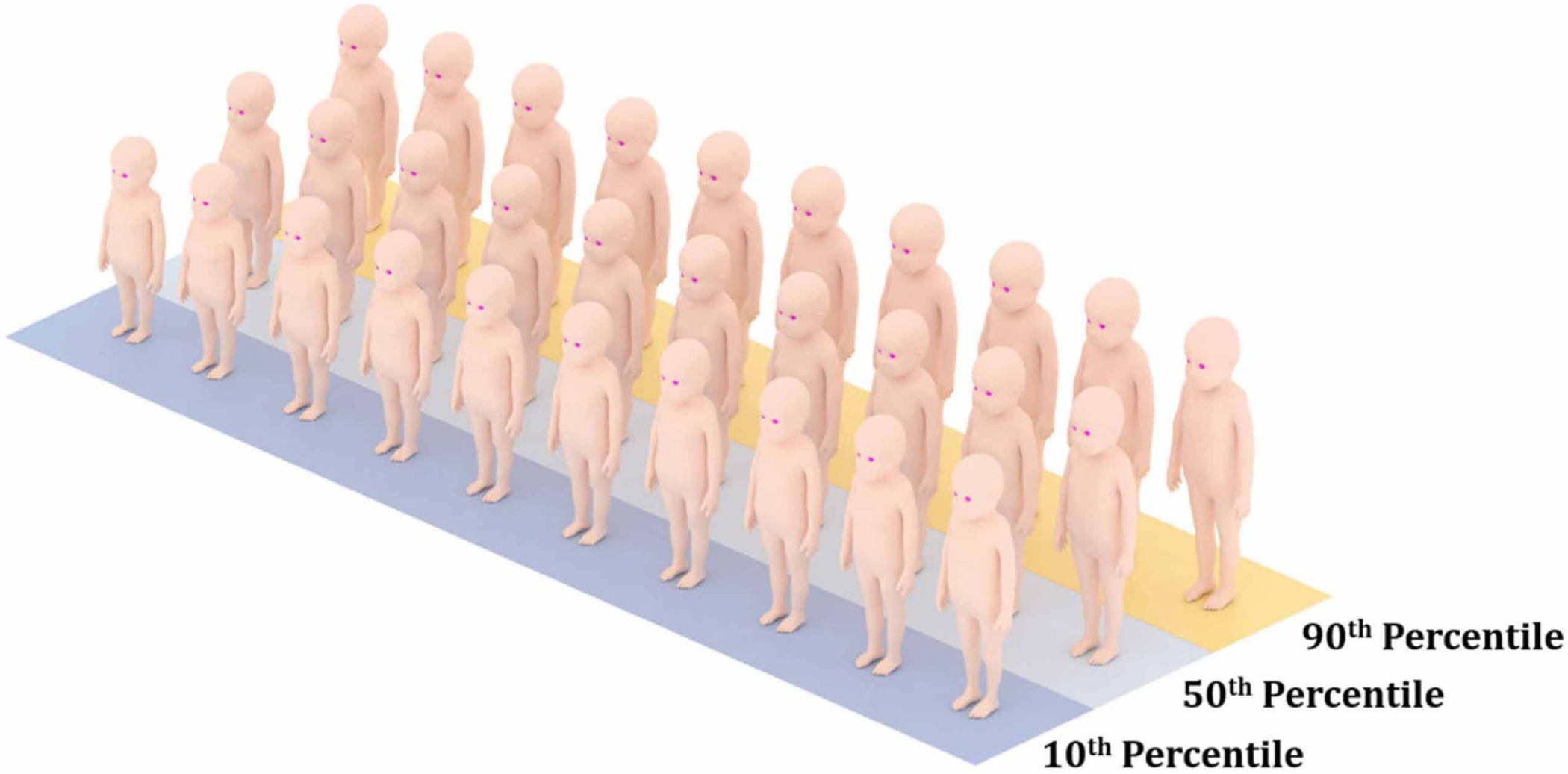
Visualization of a collection of phantoms constructed from the 1 year female MRCP. From left to right, the phantom ages range from 6 to 24 months within each percentile group.

**Figure 6.**
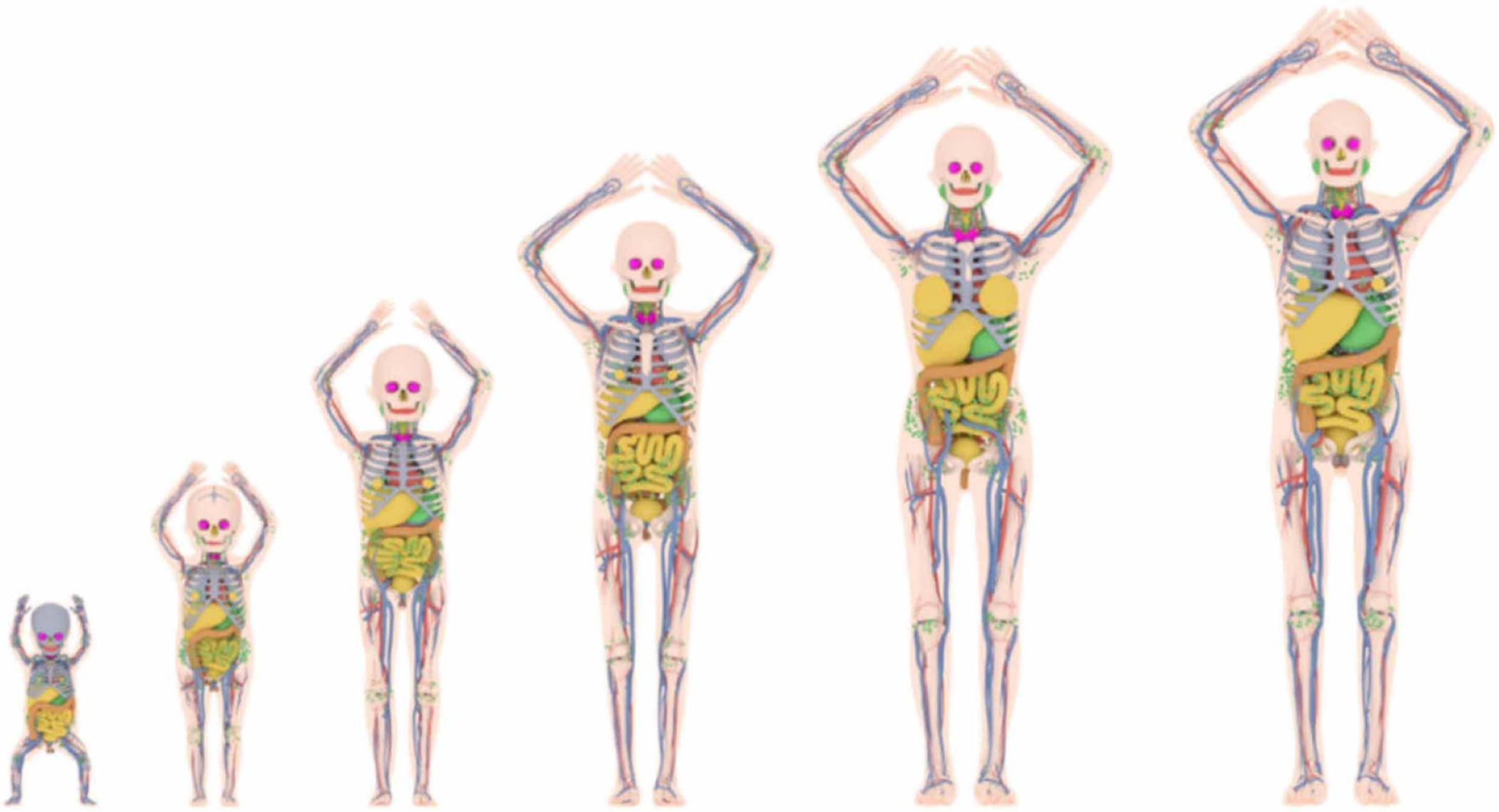
Phantoms in the AU position created using the newborn, 1-, 5-, 10-, and 15 year-old MRCPs. Both sexes are shown for the 15 year-old MRCP-derived phantoms, while only the male phantom is shown for younger ages.

Figure 4 shows the subset 15 year-old male phantoms used for the CT simulations. Figure 5 displays phantoms from the 1 year-old female MRCP at each of the percentile groups from 6 to 24 months. The AU versions of the 15 year-old male and female MRCP along with the newborn, 1 year, 5 year and 10 year male MRCPs are shown in figure 6.

### 3.3. CT dosimetry

**Figure 7.**
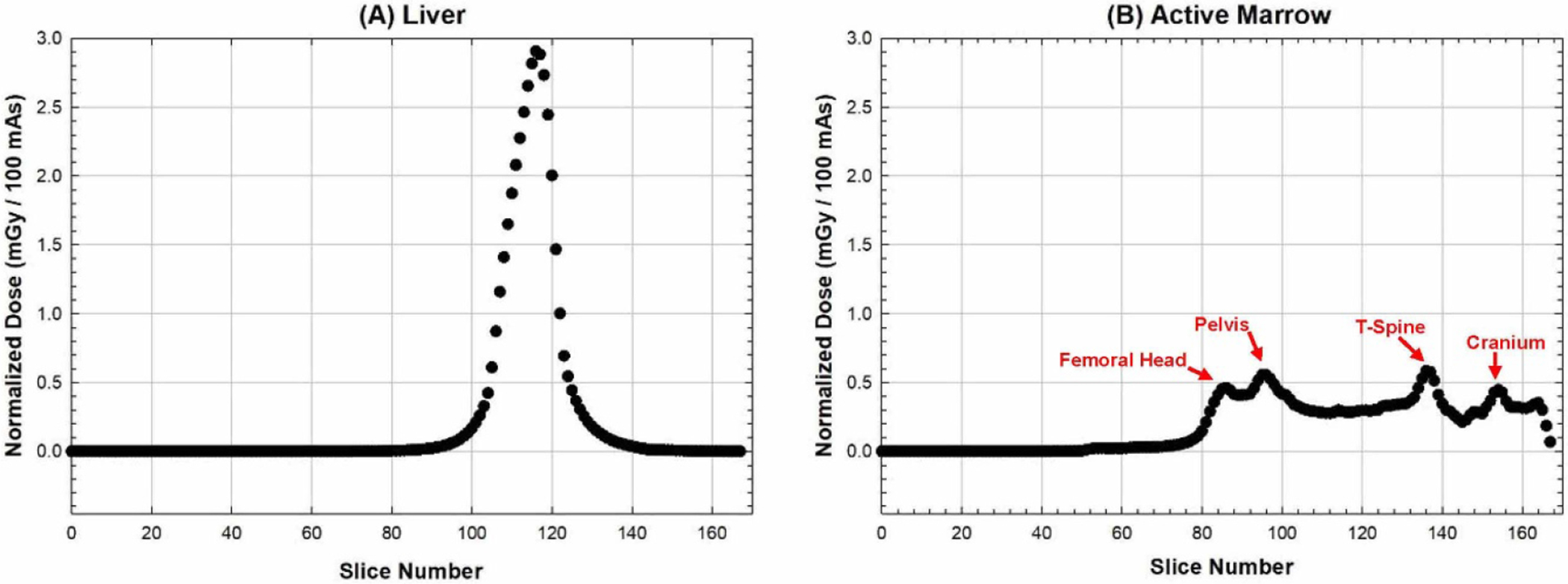
Normalized dose [mGy/100 mAs] as a function of slice number for (A) liver and (B) active marrow in the AD 15 year male MRCP.

**Figure 8.**
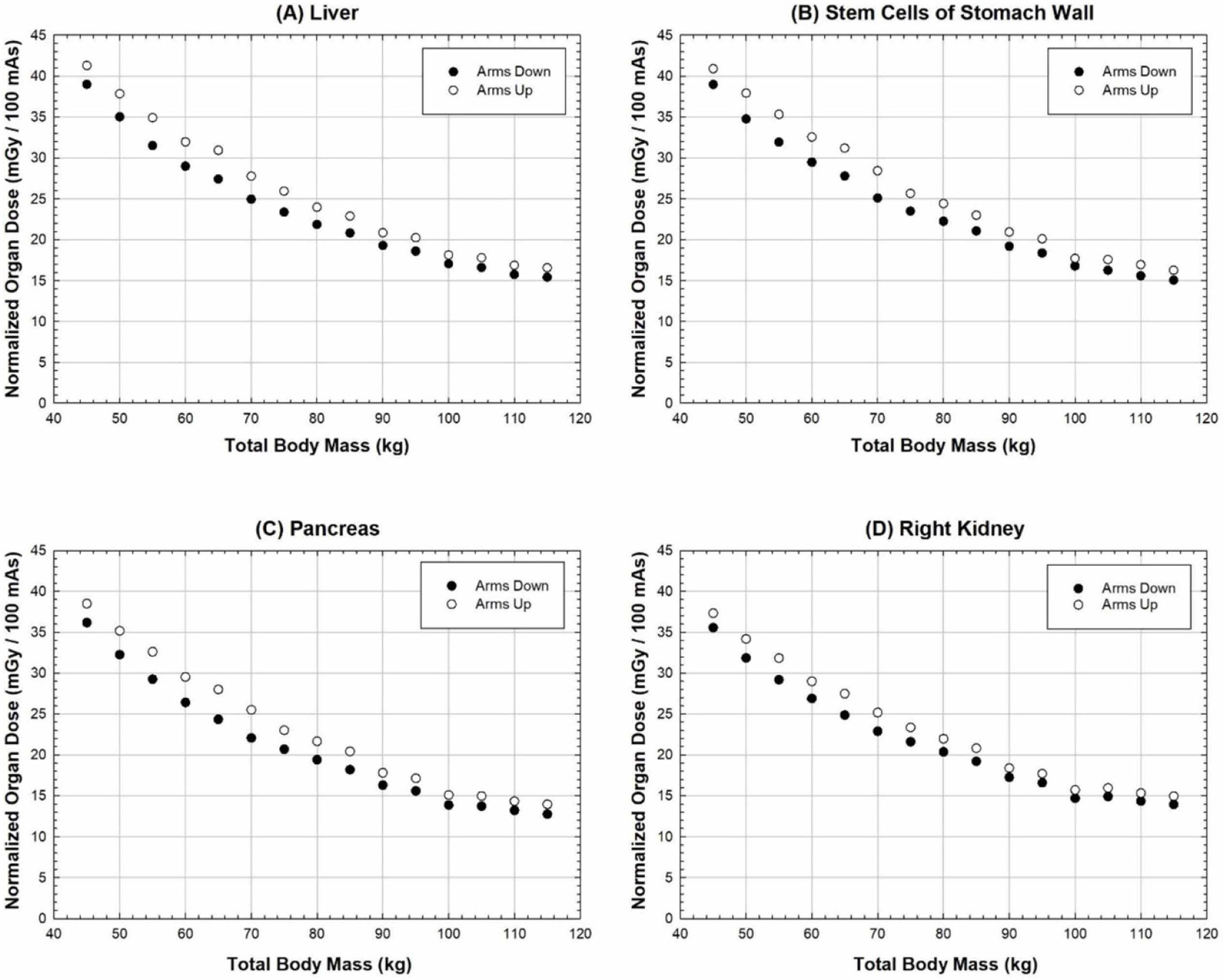
Normalized dose [mGy/100 mAs] as a function of total body mass from an abdominal scan for (A) liver, (B) stem cells of the stomach wall, (C) pancreas, and (D) right kidney, for both AD and AU versions of the 15 year male phantoms at 170 cm height.

**Figure 9.**
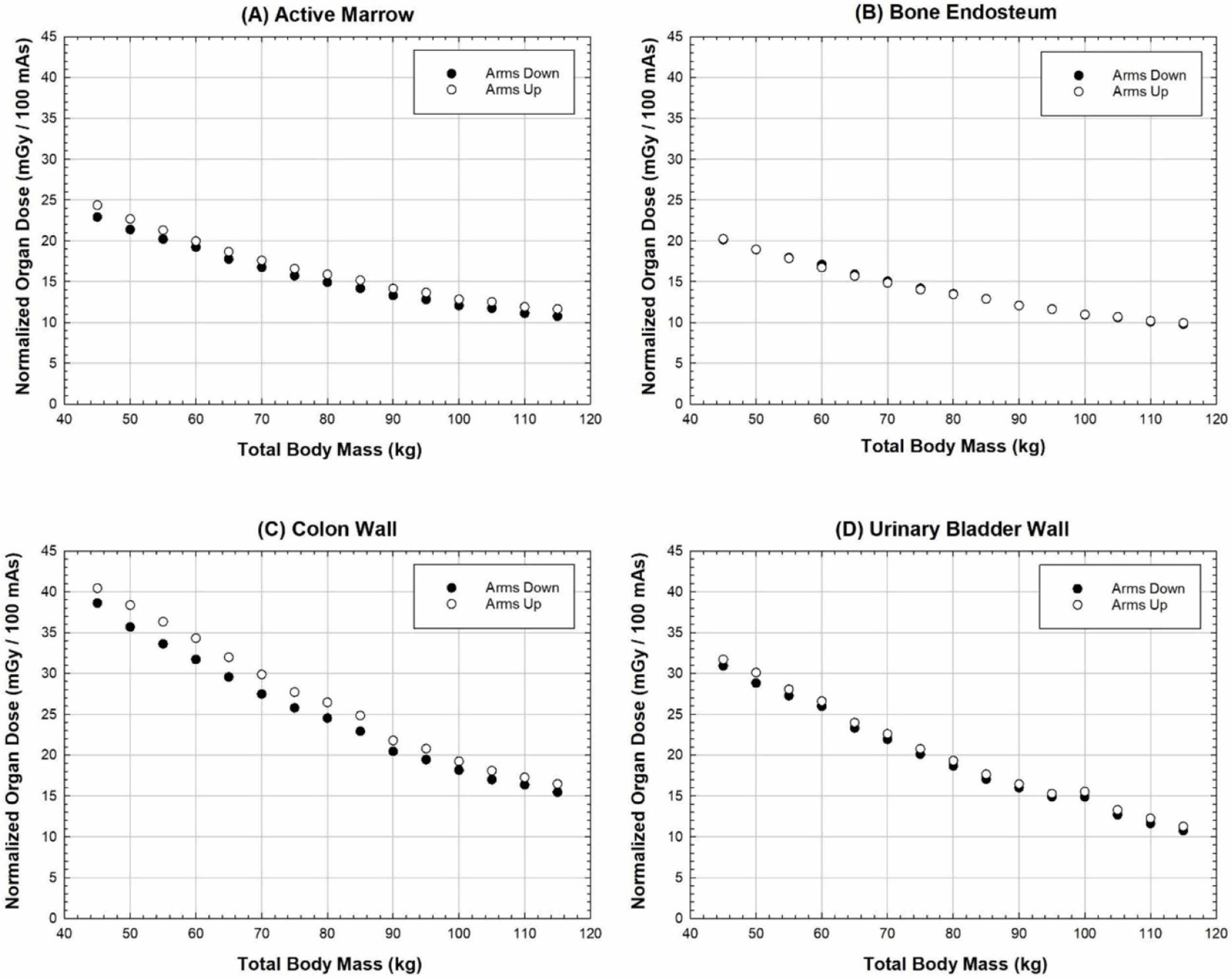
Normalized dose [mGy/100 mAs] as a function of total body mass from an chest, abdomen, pelvis (CAP) scan for (A) red (active) marrow, (B) 50 mm endosteal region, (C) colon wall, and (D) urinary bladder wall, for both AD and AU versions of the 15 year male phantoms at 170 cm height.

**Figure 10.**
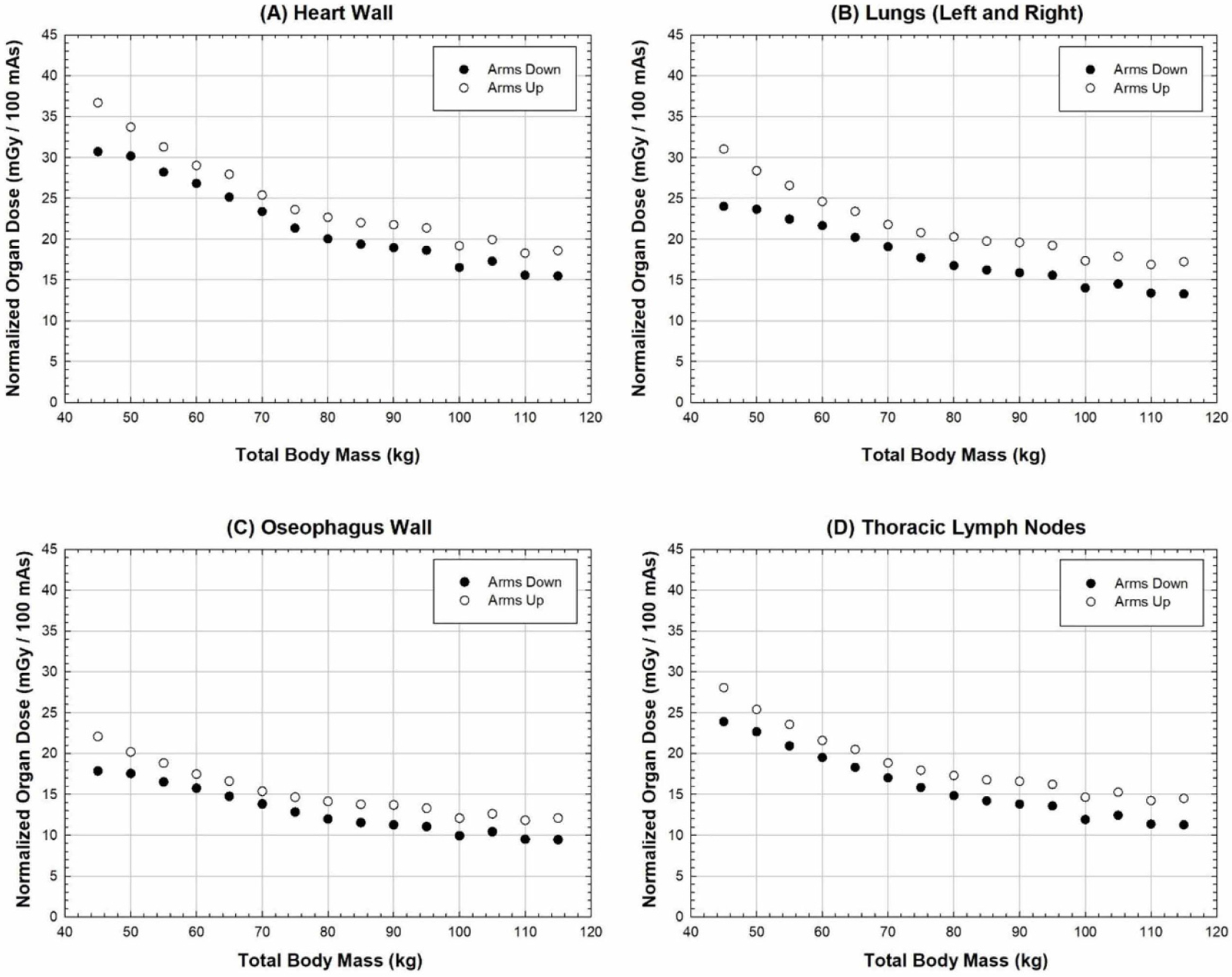
Normalized dose [mGy/100 mAs] as a function of total body mass from a cardiac scan for (A) heart wall, (B) left and right lung, (C) esophagus wall, and (D) lymph nodes within the thoracic cavity, for both AD and AU versions of the 15 year male phantoms at 170 cm height.

Normalized organ absorbed dose values for the liver and active bone marrow are plotted as a function of slice number for the AD 15 year male MRCP in figure 7. A direct relationship can be observed between dose values and the percentage of the target region’s volume within a given slice. These slices are 1 cm in thickness and the numbering of slices begins in the bottom of the feet (slice 0), and progresses to the crown of the head. Several total organ doses were computed by the summation of slice-specific organ values for a subset of the 15 year male phantoms at 170 cm SH for abdomen, chest-abdomen-pelvis (CAP) and cardiac scans as defined by pediatric protocols provided by UF SNIPs as shown in figures 8–10. Both of the simulation results from the 15 year MRCP and 170 cm variants were computed using a 120 kVp beam, 20 mm beam collimation, and medium bowtie filter under a fixed tube current scanning protocol.

## 4. Discussion

This work resulted in one of the most extensive collections of mesh-based pediatric computational phantoms, spanning a wide range of ages and body sizes. One of the major strengths of this library is its extensive representation of patients younger than two years of age, a population that is underrepresented in most existing pediatric phantom collections. This capability is especially important for pediatric dosimetry applications such as fluoroscopically guided interventions and CT imaging of infants with congenital heart disease, where patient size strongly influences absorbed dose.

For all phantoms, organ, tissue, and regional naming conventions were maintained in accordance with the MRCPs. Non-NIT phantoms follow the naming scheme used in the UF/MSK adult library, while NIT phantoms are identified using age in months combined with percentile-based anthropometric metrics. Within a given height series, variation in TBM was primarily achieved through adjustments to the subcutaneous adipose layer, with minor differences in underweight phantoms resulting from axial scaling. It should also be noted that for the 15 year-old female phantoms, breast volume was varied with BMI while maintaining constant glandular-to-adipose tissue ratio. Although pediatric breast composition is known to vary, these effects were not modeled in the present work and may be incorporated in future iterations given the radiosensitivity of breast tissue.

**Figure 11.**
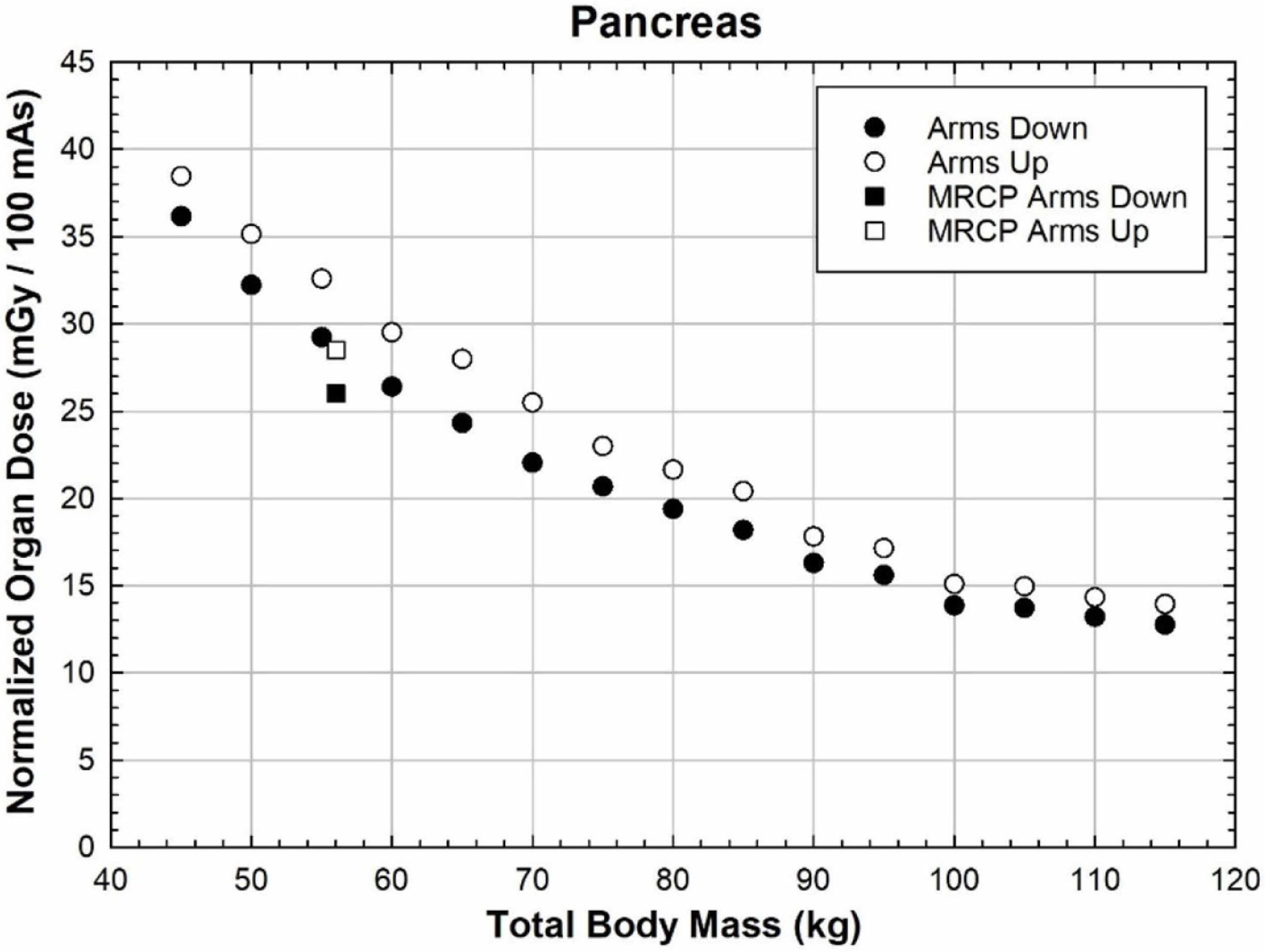
Normalized dose (mGy/100 mAs) of the pancreas as a function of total body mass from an abdominal scan for both AD and AU versions of the 15 year male MRCP and 170 cm variants.

For CT dosimetry, comparisons between AD and AU phantoms for a given height series showed modestly higher organ dose values for AU phantoms which may be attributed to additional shielding of the torso by the presence of the arms in the AD positioning (figure 11). These results assume fixed tube current, however in clinical practice, tube current modulation is commonly used and may reduce dose differences between arm positions due to changes in attenuation and mAs. Additional discrepancies in dosimetric trends may also be attributed to minor mesh artifacts created from repositioning of the arms that were later corrected by manual techniques.

Finally, comparisons with the corresponding ICRP reference phantoms demonstrate substantial differences in organ dose estimates, highlighting the importance of libraries that span realistic pediatric morphometries. The UF/MSK pediatric phantoms will be integrated into the MIRDct platform and will support CT as well as photon and proton therapy dosimetry studies (Ramos et al 2026, Dinwiddie et al 2026), enabling more accurate and patient-specific dose assessments.

## 5. Conclusion

The UF/MSK pediatric library uses state-of-the-art computational phantoms to create a collection of 59 male and 59 female models representing pediatric ages from the newborn to the 2 year-old, as well as 153 male and 131 female phantoms modeling the pediatric patient across the age range of 2 years to 18 years of age. Each AD phantom has a companion version in the arms-up position, thus addressing clinical CT scenarios and enabling more realistic organ dosimetry to create the largest grouping of pediatric computational phantoms presently available in both polygon and tetrahedral mesh formats. All anthropometric parameters were derived from data published on United States children and covers an extensive range of body morphometries. An initial collection of phantoms was simulated via MC methods to create a slice-specific organ dose library for CT dosimetry using a validated user defined source term on a Canon Aquilion One Genesis scanner. Further simulations will be performed on remaining pediatric phantoms to generate a complete database to be integrated into the freely available MIRDct software as a part of the broad suite of dosimetric tools provided at MIRDsoft.org. The UF/MSK pediatric library advances the field of medical physics by providing the first extensive mesh-type collection tailored to U.S. demographics, offering unparalleled flexibility for tetrahedralization, MC simulations, and scalable applications in diagnostic radiology, nuclear medicine and radiotherapy. When combined with the companion adult series, it forms a comprehensive, age-inclusive resource for organ dosimetry studies.

## Supplementary Material

Supplementary material for this article is available online

## Figures and Tables

**Table 1.** Mapping of reference phantoms and direction of standing height scaling for each standing height series. The direction of the arrow indicates either an increase or decrease in standing height from the MRCP source phantom.

Phantom standing height (cm)	Non-NIT males	Non-NIT females
185	MRCP-15M 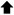	
180	MRCP-15M 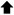	
175	MRCP-15M 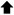	MRCP-15F 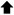
170	MRCP-15M 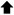	MRCP-15F 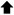
165	MRCP-15M 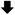	MRCP-15F 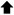
160	MRCP-15M 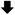	MRCP-15F 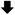
155	MRCP-15M 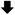	MRCP-15F 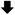
150	MRCP-10M 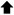	MRCP-15F 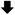
145	MRCP-10M 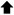	MRCP-10F 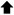
140	MRCP-10M 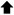	MRCP-10F 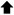
135	MRCP-10M 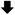	MRCP-10F 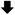
130	MRCP-10M 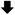	MRCP-10F 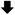
125	MRCP-10M 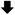	MRCP-10F 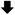
120	MRCP-05M 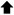	MRCP-05F 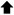
115	MRCP-05M 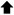	MRCP-05F 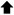
110	MRCP-05M 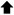	MRCP-05F 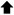
105	MRCP-05M 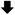	MRCP-05F 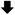
100	MRCP-05M 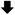	MRCP-05F 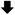
95	MRCP-05M 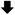	MRCP-05F 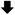

## Data Availability

The data of the study will be made available upon request of the authors. PHITS radiation transport files for all Monte Carlo radiation transport simulations conducted herein will be made available at a publicly accessible GitHub repository. All data that support the findings of this study are included within the article (and any supplementary information files). Annex_A_Parameters_Ped_Males available at https://doi.org/10.1088/1361-6560/ae4a81/data1. Annex_B_Parameters_Ped_Females available at https://doi.org/10.1088/1361-6560/ae4a81/data2. Annex_C_Parameters_NIT available at https://doi.org/10.1088/1361-6560/ae4a81/data3. Annex_D_OrganMass_Ped_Males available at https://doi.org/10.1088/1361-6560/ae4a81/data4. Annex_E_OrganMass_Ped_Females available at https://doi.org/10.1088/1361-6560/ae4a81/data5. Annex_F_OrganMass_NIT available at https://doi.org/10.1088/1361-6560/ae4a81/data6.

## Footnotes

9 https://www.cdc.gov/growthcharts/cdc-growth-charts.htm.

10 https://protocols.xray.ufl.edu/live_protocols/snips/.
